# RACIAL/ETHNIC AND MULTIMORBIDITY DIFFERENCES IN DEMENTIA ONSET AMONG MIDDLE-AGED AND OLDER ADULTS

**DOI:** 10.1093/geroni/igad104.2416

**Published:** 2023-12-21

**Authors:** Siting Chen, Corey Nagel, Anda Botoseneanu, Heather Allore, Jason Newsom, Stephen Thielke, Jeffrey Kaye, Ana Quiñones

**Affiliations:** Oregon Health & Science University, Portland, Oregon, United States; University of Arkansas for Medical Sciences, Little Rock, Arkansas, United States; University of Michigan, Ann Arbor, Michigan, United States; Yale University, New Haven, Connecticut, United States; Portland State University, Portland, Oregon, United States; University of Washington, Seattle, Washington, United States; Oregon Health & Science University, Portland, Oregon, United States; Oregon Health & Science University, Portland, Oregon, United States

## Abstract

Racial/ethnic minoritized older adults in the U.S. have higher prevalence of multimorbidity and experience higher risk of dementia relative to non-Hispanic White older adults. This study examined the relationship between race/ethnicity and dementia onset within the context of multimorbidity during middle and later life in the U.S. Data from the Health & Retirement Study (1998-2018, HRS and War Babies cohorts, N=8,031) were applied to discrete-time survival models to estimate differences in the risk of dementia onset using the Langa-Weir Classification for up to ten time intervals (1998-2018). Models were adjusted for race/ethnicity (non-Hispanic White, non-Hispanic Black, Hispanic), multimorbidity (no/one disease, advanced cardiovascular multimorbidity, metabolic multimorbidity, advanced cardiovascular-metabolic multimorbidity, and neither advanced cardiovascular nor metabolic multimorbidity), age at baseline, sex, education, and whether a proxy provided the interview. Over the 20 years, 9.8% of the respondents (n=785) developed dementia. In covariate-adjusted models, non-Hispanic Black (adjusted Odds Ratio (aOR) =2.27, 95%CI: 1.90, 2.72) and Hispanic (aOR=1.59, 95%CI: 1.26, 2.01) respondents had higher risk of developing dementia compared with non-Hispanic White respondents. Regarding multimorbidity categories, respondents with advanced cardiovascular-metabolic multimorbidity (aOR=1.80, 95%CI: 1.45, 2.24) had significantly higher odds of dementia onset relative to the neither advanced cardiovascular nor metabolic multimorbidity category. Significant differences were observed in dementia onset for up to 20 years between racial/ethnic minoritized and White adults, as well as among multimorbidity combinations. Our findings highlight the need to intervene earlier on modifiable risk factors, such as multimorbidity, to reduce the risk of developing dementia among racial/ethnic minoritized adults.

